# Supplementary material for: Cost-effectiveness of mechanical thromboprophylaxis for cesarean deliveries in Brazil
Source: PLoS One. 2023 Jun 29;18(6):e0287812. doi: 10.1371/journal.pone.0287812 (PMC10309987; doi:10.1371/journal.pone.0287812)
Supplement: S1 File — (DOCX) [file pone.0287812.s001.docx]

Supporting information: Cost-effectiveness of mechanical thromboprophylaxis for cesarean deliveries in Brazil

Alex Veloz, Ubong Silas, Rhodri Saunders, Jody Grisamore, André Luis Malavasi


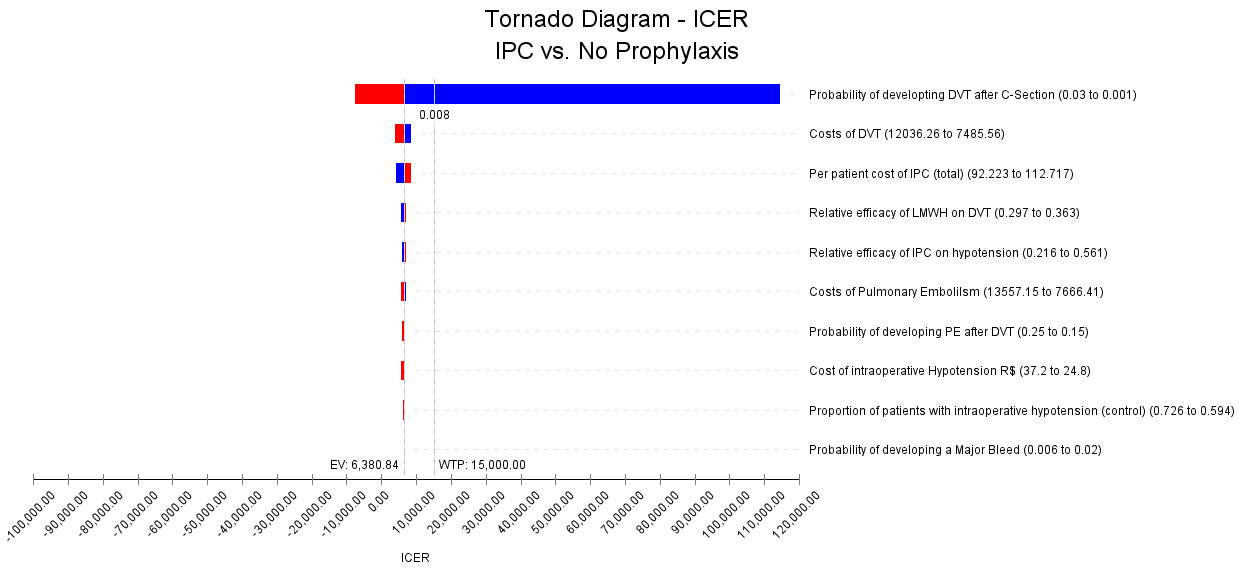


Fig. S1 One-way sensitivity analysis of IPC versus “No prophylaxis” showing the top ten influential parameters affecting the ICER. ICER, incremental cost-effectiveness ratio; DVT, deep vein thrombosis; C-section, cesarean section; IPC, intermittent pneumatic compression; LMWH, low-molecular-weight heparin; PE, pulmonary embolism, R$; Brazilian Real


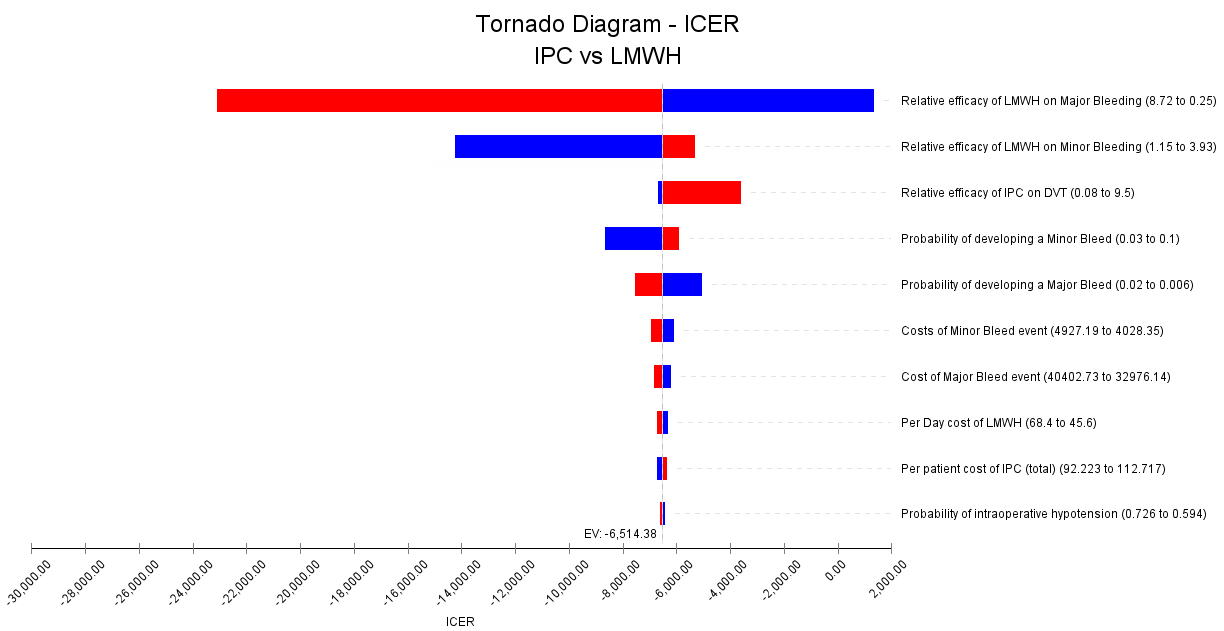


Fig. S2 One-way sensitivity analysis of IPC versus LMWH showing the top ten influential parameters affecting the ICER. ICER, incremental cost-effectiveness ratio; DVT, deep vein thrombosis; C-section, cesarean section; IPC, intermittent pneumatic compression; LMWH, low-molecular-weight heparin; PE, pulmonary embolism, R$; Brazilian Real

# CHEERS Checklist: reporting standards for health economic evaluations

Table 1 provides the Consolidated Health Economic Evaluation Reporting Standards (CHEERS 2022) checklist ^1^ with references to page and line numbers in the original manuscript.

Table S1 CHEERS 2022 checklist: reporting standards for health economic evaluations

| **Selection/Item** | **Item** | **Recommendation** | **Page /Line*** |
| --- | --- | --- | --- |
| Title and abstract | | | |
| Title | 1 | Identify the study as an economic evaluation and specify the interventions being compared. | 1/ 2-3 |
| Abstract | 2 | Provide a structured summary that highlights context, key methods, results, and alternative analyses. | 2/ 17-41 |
| Introduction | | | |
| Background and objectives | 3 | Give the context for the study, the study question, and its practical relevance for decision making in policy or practice. | 3/ 42-96 |
| Methods | | | |
| Health economic analysis plan | 4 | Indicate whether a health economic analysis plan was developed and where available. |  |
| Study population | 5 | Describe characteristics of the study population (such as age range, demographics, socioeconomic, or clinical characteristics). | 5/ 104-110 |
| Setting and location | 6 | Provide relevant contextual information that may inﬂuence ﬁndings. | 5/ 98-101 |
| Comparators | 7 | Describe the interventions or strategies being compared and why chosen. | 6/ 132-136 |
| Perspective | 8 | State the perspective(s) adopted by the study and why chosen. | 5/ 98-99 |
| Time horizon | 9 | State the time horizon for the study and why appropriate. | 5/100-101 |
| Discount rate | 10 | Report the discount rate(s) and reason chosen. | 5/116-117 |
| Selection of outcomes | 11 | Describe what outcomes were used as the measure(s) of beneﬁt(s) and harm(s). | 7/144-148 |
| Measurement of outcomes | 12 | Describe how outcomes used to capture beneﬁt(s) and harm(s) were measured. | 7/144-148 |
| Valuation of outcomes | 13 | Describe the population and methods used to measure and value outcomes. | 7/144-148 |
| Measurement and valuation of resources and costs | 14 | Describe how costs were valued. | 7/153-160 |
| Currency, price date, and conversion | 15 | Report the dates of the estimated resource quantities and unit costs, plus the currency and year of conversion. | 7/158-159 |
| Rationale and description of model | 16 | If modeling is used, describe in detail and why used. Report if the model is publicly available and where it can be accessed. | 6/ 130-138 |
| Analytics and assumptions | 17 | Describe any methods for analysing or statistically transforming data, any extrapolation methods, and approaches for validating any model used. | 9/173-187 |
| Characterizing heterogeneity | 18 | Describe any methods used for estimating how the results of the study vary for subgroups. | 10/199-200 |
| Characterizing distributional effects | 19 | Describe how impacts are distributed across different individuals or adjustments made to reﬂect priority populations. |  |
| Characterizing uncertainty | 20 | Describe methods to characterize any sources of uncertainty in the analysis. | 10/ 188-200 |
| Approach to engagement with patients and others affected by the study | 21 | Describe any approaches to engage patients or service recipients, the general public, communities, or stakeholders (such as clinicians or payers) in the design of the study. |  |
| Results | | | |
| Study parameters | 22 | Report all analytic inputs (such as values, ranges, references) including uncertainty or distributional assumptions. | 8/ 165-171 |
| Summary of main results | 23 | Report the mean values for the main categories of costs and outcomes of interest and summarize them in the most appropriate overall measure. | 10/ 202-209 |
| Effect of uncertainty | 24 | Describe how uncertainty about analytic judgments, inputs, or projections affect ﬁndings. Report the effect of choice of discount rate and time horizon, if applicable. | 11/213-249 |
| Effect of engagement with patients and others affected by the study | 25 | Report on any difference patient/service recipient, general public, community, or stakeholder involvement made to the approach or ﬁndings of the study. |  |
| Discussion | | | |
| Study ﬁndings, limitations, generalisability, and current knowledge | 26 | Report key ﬁndings, limitations, ethical or equity considerations not captured, and how these could affect patients, policy, or practice. | 13/ 251-313 |
| Other relevant information | | | |
| Source of funding | 27 | Describe how the study was funded and any role of the funder in the identiﬁcation, design, conduct, and reporting of the analysis. |  |
| Conﬂicts of interest | 28 | Report authors conﬂicts of interest according to journal or International Committee of Medical Journal Editors requirements. |  |

References

1. Consolidated Health Economic Evaluation Reporting Standards (CHEERS) 2022 Explanation and Elaboration: A Report of the ISPOR CHEERS II Good Practices Task Force. *Value Health*. 2022;25(6):1060. doi:10.1016/j.jval.2022.03.002.
